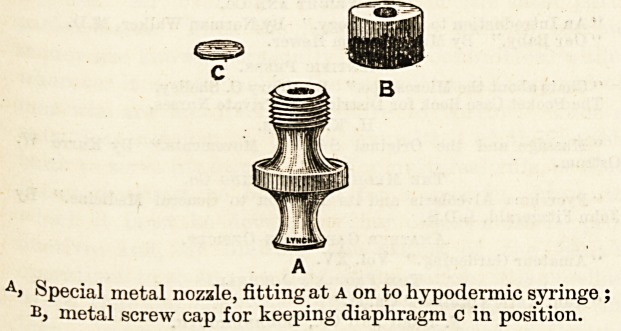# New Appliances and Things Medical

**Published:** 1899-07-08

**Authors:** 


### NEW APPLIANCES AND THINGS MEDICAL.

[We shall be glad to receive, at our Office, 28 & 29, Southampton Street, Strand, London, W.C., from the manufacturers, specimens of all new preparations and appliances which may be brought out from time to time.]

### NEW EJECTOR FOR VACCINE LYMPH.

(Lynch and Co., 192, Aldersgate Street, E.C.)

This is a most useful invention for the expulsion or sucking-up of vaccine lymph, and a safe substitute for the mouth, the usual medium among medical practitioners. The ejector is composed entirely of metal, except for a small diaphragm, which is perforated in the centre for the reception of the tube. This diaphragm may be either of india-rubber or leather. The metal mount is so arranged that it can be fixed in the nozzle of an ordinary hypodermic syringe, the piston of the syringe expelling or sucking-up the lymph according as it is lowered or raised. The simplicity of the invention, its durability, and the price, *i.e.*, 1s., should secure for itself popularity among practitioners and public vaccinators.

A, Special metal nozzle, fitting at A on to hypodermic syringe; B, metal screw cap for keeping diaphragm C in position.

### NEW WOOD BLOCK FLOORING.

(Thomas Charteris and Co., 31 and 32, King William Street, E.C.)

The above firm have now perfected a system of wood block flooring, which, both from an artistic and practical point of view, is infinitely superior to the old method of plain blocks. Every block is held in position by a special system of dovetailing, and the entire flooring is fixed to the substructure by means of specially-prepared mastic composition. It is thus impossible to move any single block, when once in position, without removing the whole floor. Provision is, however, made for obtaining easy access to hot-water, gas, and other pipes by means of an ingenious device, which is called the special "B" system. For hospital wards, dormitories, and the like this special block flooring has the supreme advantage that it may be cleaned with hose and squeegee, in which case, however, the ordinary skirting must be replaced with special Scotia fillets. Among other advantages of this flooring for hospitals and institutions may be mentioned its economy, noiselessness, fire and damp resisting qualities, and, when properly laid, the greater amount of head room and loftiness of ceiling, which it ensures.

## Figures and Tables

**Figure f1:**